# Natural Killer Cell Integrins and Their Functions in Tissue Residency

**DOI:** 10.3389/fimmu.2021.647358

**Published:** 2021-03-10

**Authors:** Michael J. Shannon, Emily M. Mace

**Affiliations:** Department of Pediatrics, Vagelos College of Physicians and Surgeons, Columbia University, New York, NY, United States

**Keywords:** NK cell, integrin, adhesion, cell migration, tissue residency

## Abstract

Integrins are transmembrane receptors associated with adhesion and migration and are often highly differentially expressed receptors amongst natural killer cell subsets in microenvironments. Tissue resident natural killer cells are frequently defined by their differential integrin expression compared to other NK cell subsets, and integrins can further localize tissue resident NK cells to tissue microenvironments. As such, integrins play important roles in both the phenotypic and functional identity of NK cell subsets. Here we review the expression of integrin subtypes on NK cells and NK cell subsets with the goal of better understanding how integrin selection can dictate tissue residency and mediate function from the nanoscale to the tissue environment.

## Introduction

**Figure 1:**
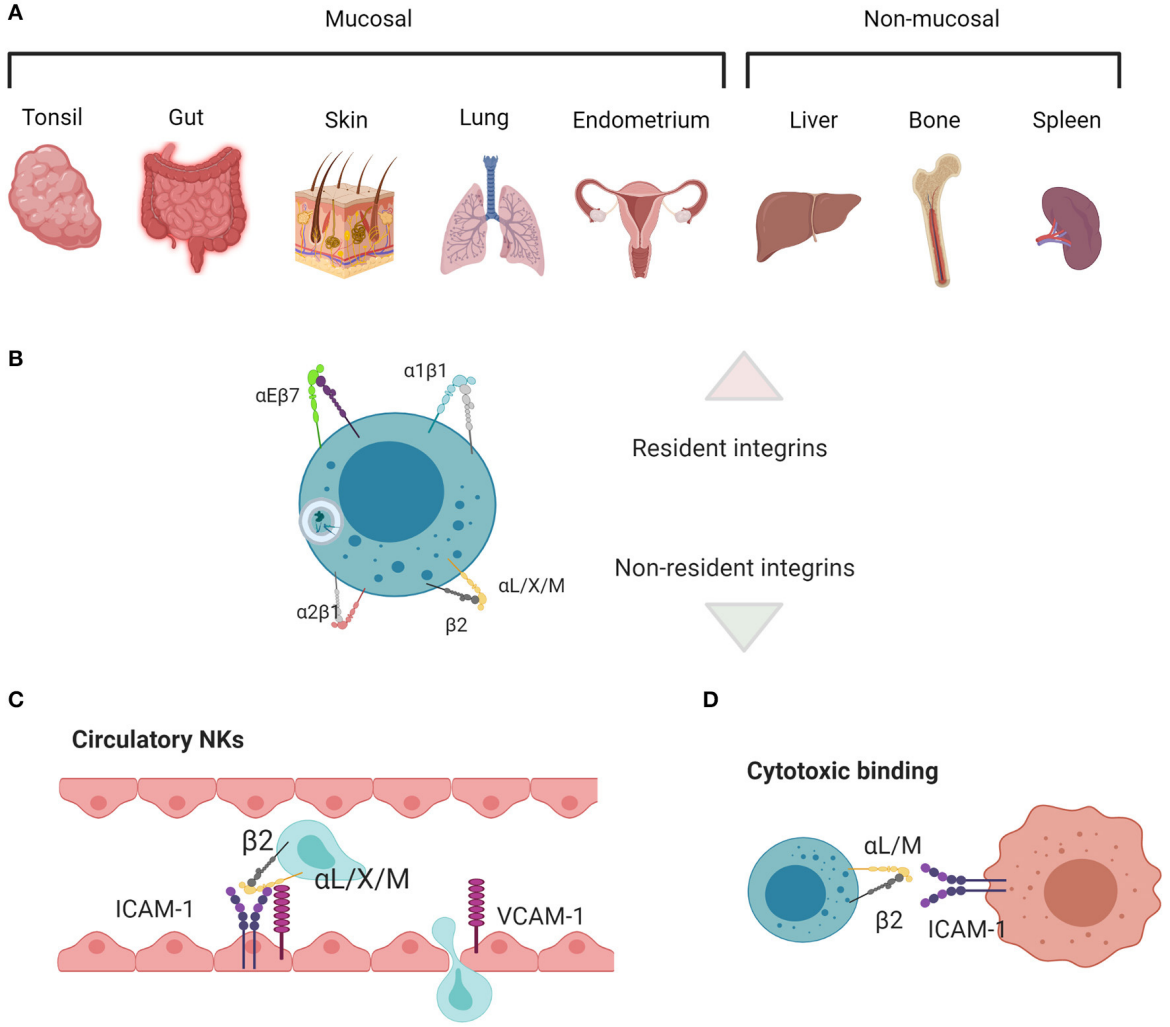
Main tissue resident vs. non-resident integrin functions in NK cells. **(A)** Mucosal and non-mucosal tissues in which NK cell residence has been investigated. **(B)** αEβ7 and α1β1 are associated with residency, whereas α2β1 and the β2 integrins are associated with migration, adhesion, and cytotoxicity. **(C)** β2 integrins enable cells to migrate in and out of circulation and through tissues, along with **(D)** mediating adhesion and signaling during target cell lysis. Created with BioRender.com.

NK cell subsets are defined by surface receptors, especially integrins, transcription factors, and intracellular effector molecules. There is still much to learn about how tissue resident and circulating NK cells are generated and undergo specialization for functions including cytotoxicity and cytokine production. However, the classification of many tissue resident NK cell subsets by the expression of integrins, including α1 and αE integrins, when compared with non-resident cells that primarily express β2 integrins, is evidence for the relevance of integrins to environmental adaptation in addition to developmental and functional processes (Figure 1). To add to the complexity of the use of integrins as phenotypic parameters, multiple levels of nomenclature for integrins on immune cells exist, occasionally masking the true nature of many of the cluster of differentiation (CD) molecules that are used to define NK cell subsets as integrin subunits.

Integrins communicate signals to and from the extracellular matrix (ECM) and other cells. Integrin ligands include ECM components, including 20 different isoforms of fibronectin and multiple collagen and laminin family members, plus selectins, and cell adhesion molecules (CAMs) (1). Integrins form nanometer-scale signaling islands which are regulated by chemokine receptors and can be defined as clusters of integrins, adaptors, scaffolds, kinases, phosphatases, and actin linkers. Such signaling clusters have also been described as “nano-adhesions” in lymphocytes, which form functional units to send signals cell-wide and influence cell behavior (2). Lymphocyte nano-adhesions have the same size and many of the same molecules as nascent adhesions in non-lymphocytes (3), but have shorter lifetimes due to much faster cell migration and adhesion turnover. Each cell exists as part of a highly heterogenous 3D population of cells, all of which can influence each other to produce a cohesive, multi-faceted, system-level response. Integrin communication clusters are at the heart of complex signaling axes that include chemokine receptors and tissue architecture and mediate the homing and tissue residency of immune cells (4).

The use of CD nomenclature has simplified the definition of immune subsets but can also obscure the biological nature of the markers that are represented. Interpretation of genetic data related to integrins is limited by our inability to discern heterodimers. Individual β subunits form heterodimers with divergent functions depending on the α subunit that they are paired with. Antibody detection, with a few notable exceptions for those that recognize unique epitopes only present in the heterodimeric form, similarly only provides information about single subunits (5). Therefore, while ample data, particularly from flow cytometry, offers us information about the phenotype of tissue resident NK cell subsets, we still lack a comprehensive understanding of how cells become specialized to their microenvironment, what dictates their residency, and the relationship between heterodimeric integrin expression and their spatial localization and function. This knowledge gap also highlights the need for cell biological analyses that offer answers correlatively across time and length scales.

Here, we summarize integrin expression on NK cell subsets in humans and mice and ask what we can infer from the functions of integrins in relation to what is known about the tissue environments that support NK cell development and residency. By considering the single molecule functions of integrin subunits and heterodimers, we aim to integrate what is known about the likely function of integrins in different NK cell populations. Further, we discuss key outstanding questions surrounding the relative effects of inherent integrin phenotypes and plasticity in response to environmental cues. Finally, we summarize techniques which will be important for future multiscale investigations of integrins in heterogeneous tissue resident NK cell populations.

## Introduction to Integrin Structure and Function

Integrins in immunology are often described as single subunits as they are detected this way by flow cytometry, RNA-Seq and proteomic analyses. Such data are unmatched for analyzing single cells in heterogeneous populations and have driven much of our underlying knowledge of immune cell phenotypes. However, integrins are obligate heterodimers composed of an α and a β subunit, so we must start here if we are to investigate their wider function.

There are 18 known integrin α subunits and 8 β subunits, and each α subunit can differentially pair with multiple β partners to generate 24 known integrin heterodimers (6). The mix of integrins on a single cell, or group of cells, has been described as an “area code,” a unique signature that directs cells to home to a particular locale and can help them perform a more generalized task, such as adhesion, migration, diapedesis, proliferation, survival, or residency (7–9). While the same integrin heterodimer can have multiple extracellular binding partners, including collagen, laminin, fibronectin, and the CAM family of adhesion receptors, these binding partners may have unique binding sites on different integrins. In addition to their diversity of ligand binding, integrins respond to intracellular and extracellular activating signals through rapid conformational change (10–12). Further, integrins are also mechanosensitive and participate in catch-bonding, meaning that force will modulate their structure, binding, and downstream signaling (13–15). Integrins also form clusters in the membrane, which change in size as cells alter their speed (T cells) (2). Therefore, both the structure and function of integrins are highly modular and adaptable, and we can consider integrin function collectively within the context of single molecule structure, bound ligand structure, and nanocluster-scale behavior. There are many open questions about how biology across these scales is linked. New quantitative microscopy techniques will be key to providing direct links between the collective behavior of a heterogeneous system of cells and nanoscale events that shape single cell responses.

### Integrin Protein Structure Functionally Links With Affinity and Clustering

Integrins have been extensively structurally defined and contain an ectodomain, transmembrane domain, and cytoplasmic domain that form a shape analogous to a “head” on two “legs” (16). The ectodomain binds extracellular ligand, whereas the relatively short cytoplasmic domains bind adaptors that form signaling hubs and link integrins to the actin cytoskeleton (16). Thus, integrins mediate bidirectional signaling across the cell membrane between the actin cytoskeleton and the extracellular environment. The coupling mechanism between integrins and actin is termed the “molecular clutch” owing to the dynamism of the actin cytoskeletal network and the transient binding of integrin adhesions, which act together to translate power from the inside to the outside of a migrating cell (17–20). Integrins form clusters to effectively translate force and to allow cells to quickly change their direction or adhesive behavior. In lymphocytes, integrin clusters are very small (<100 nm) and exhibit fast turnover during migration and spreading (2, 10, 17). Nonetheless, they contain many of the same adhesion components as early or nascent adhesions in fibroblasts (3, 21–23). The status of cell-wide integrin clustering is constantly in flux, on a per adhesion basis as well as between adhesions, and likely allows for effective tuning of the behavior of a given cell within its niche. The communication and crosstalk of integrin species between nano-adhesions, and the ways in which cells translate single adhesion behavior into whole cell behavior are under active study.

The directionality and mechanism of integrin signaling across the cell membrane is often referred to as inside-out vs. outside-in signaling (24, 25). Inside-out signaling pathways are initiated by other membrane receptors, particularly chemokine receptors. They result in changes to integrin affinity, the translation of forces and signals between the integrin and the inside of the cell, and the clustering of integrin heterodimers and 3D nanoscale arrangement of focal complexes. Outside-in signaling occurs as cytoplasmic adaptors link integrins to molecules that exert force within the cell, particularly the actin cytoskeleton. In addition, outside-in signaling can lead to activation of signaling cascades including the MAPK and ERK axes (26). While less well-defined, inside-in signaling occurs via endocytosed integrins which can participate in signaling to regulate gene expression (27).

Integrins adopt three affinity states according to the “switchblade model”: a low-affinity bent-closed conformation, an intermediate-affinity extended-closed conformation, and a high-affinity extended-open conformation thought only to be invoked following extracellular ligand binding (7, 28–30). αLβ2 (CD11a/CD18, LFA-1) is the best characterized integrin structurally, but electron microscopy, X-ray crystallography and NMR have confirmed similar structural changes in other integrins (31). Recent interferometric super resolution data, obtained by fluorescently tagging the membrane distal region of the α_L_ subunit, confirms that the αLβ2 heterodimer adopts both a bent and a stretched conformation in live cells and the length of the molecule changes by 16 nm upon binding to the ICAM-1 ligand (12). Crystallographic studies link this stretching with changes in affinity and demonstrate that the α_I_ domain within the α subunit of β_2_ integrin heterodimers provides higher affinity ligand binding upon allosteric interaction with an internal ligand in the stretched form (31, 32). The tightening of binding to this internal ligand stabilizes the extended-open, high affinity form to facilitate strong adhesion (32). In the case of αLβ2 integrin, often used as a model for structure/function, this transition is preceded by selectin-mediated tethering, activation by endothelium-bound chemokines, then ICAM-1 binding coupled with increased tension from shear flow in the blood (33, 34).

Most cytoplasmic β integrin tails are 40–70 residues long and are largely unstructured but form helices or pack close to the membrane depending on which intermediates they bind (35). They are moderately conserved and have binding sites that can be regulated by serine/threonine or tyrosine phosphorylation, including within the highly conserved NPxY motif (36). β cytoplasmic tails mediate binding to molecules that link integrins to the actin cytoskeleton and other signaling molecules via adaptors. α subunit cytoplasmic tails are much more structurally heterogeneous, interact with the β subunit through signaling intermediates to alter binding, and are mostly associated with specifying integrin trafficking and inhibition (35). The relationship between tertiary and quaternary protein structure caused by interactions of the β and α subunits provides specificity of binding to a given 3D motif. For example, αI and αA domains can directly bind a 6-residue collagen motif, but only form the correct structure to do this when a heterodimer is interacting with a β subunit, often in conjunction with the binding of Mn^2+^, Mg^2+^, or Ca^2+^ (16, 37). In this way, an area of cationic charge in the integrin head region is created that is specific for a structurally regulated grouping of amino acids. This enables a bond to form, the strength of which is subject to structural changes in the integrin that are transmitted across the cell membrane (35). The transmembrane regions form helices, and long-range interactions change their structure and non-covalent interaction with the surrounding lipid bilayer. Such structural changes occur due to alterations caused by ligand binding or mechanosensing in the ectodomain, or adaptor protein binding or mechanosensing on the cytoplasmic side (36).

**Figure 2:**
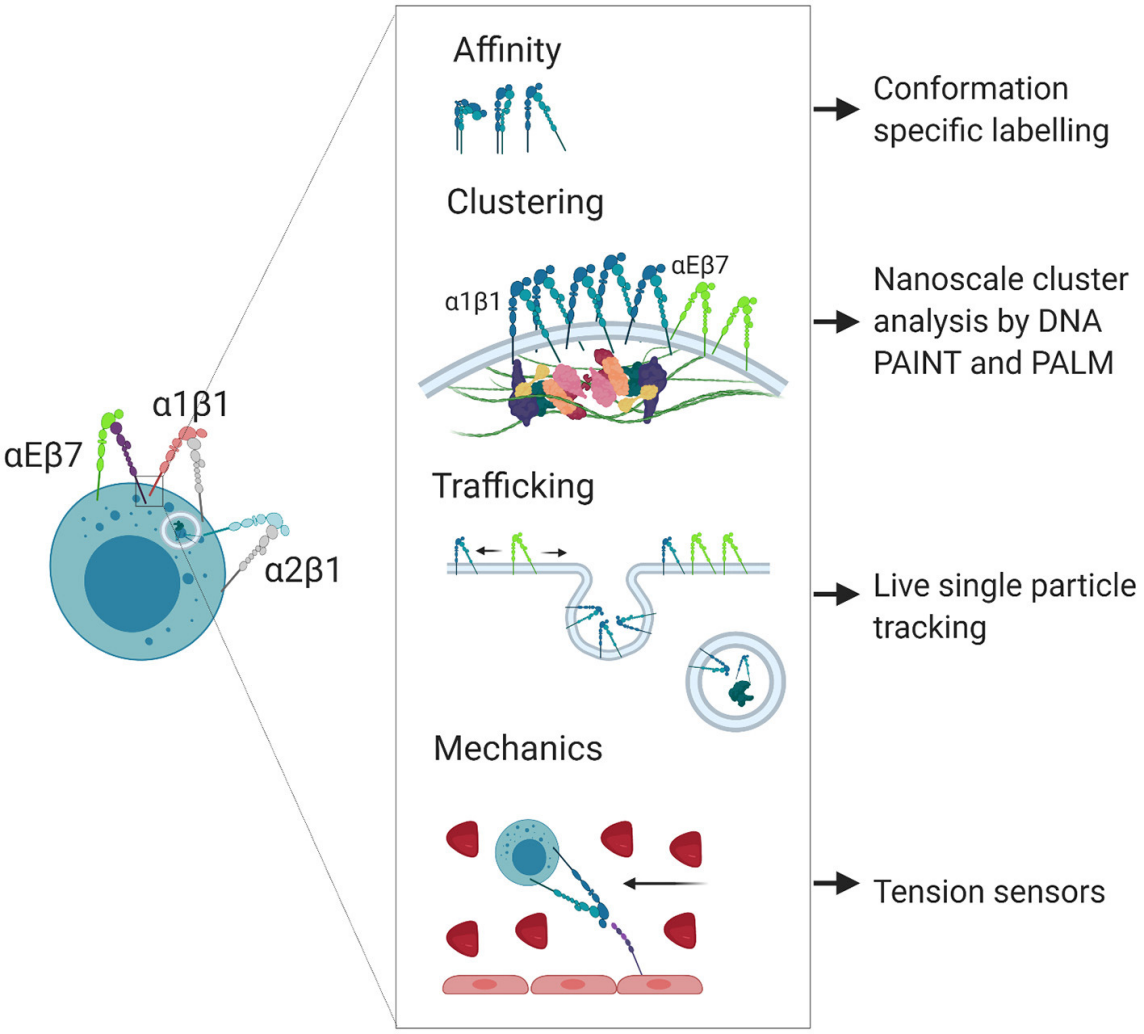
Nanoscale regulation of integrin localization and signaling. Integrins participate in poorly understood nanoscale signaling mechanisms that can be studied using super resolution microscopy techniques. Created with BioRender.com.

Integrin-ligand bond strengthening can occur as a result of mechanical stress, inducing a transition from intermediate to high affinity conformation. This strengthening can occur in response to external forces on cells following ligand binding, such as the shear flow of the blood (38, 39) or from the pulling force of other cells during immunological synapse formation (9), as well as internal force transmission through adaptor molecules to the cytoskeleton (40, 41). Bidirectional force applied following ligand binding exposes cryptic binding sites on integrin-bound talin and relieves autoinhibition of both talin and vinculin, leading to strengthening of adhesion at focal adhesion complexes (42–44). Such strengthening manifests as a coalescence of canonical integrin adhesome proteins that regulate the lifetime and clustering of adhesions (45–48). In contrast to induction of high affinity conformation, the conformational switch from low to intermediate affinity occurs due to intracellular activation through cytoplasmic regulators in response to chemokine signaling, or in response to transient binding through other ligands such as selectins (49). Rapid adhesion strengthening and weakening through catch bond or chemokine induction helps facilitate diapedesis, where cells must strengthen adhesions to resist the shear flow of the blood prior to crawling through spaces between endothelial cells, a process which may be less dependent on integrin engagement and more dependent on cytoskeletal dynamics (50, 51). The modulation of integrin affinity and the composition of adhesion clusters are also both important for tuning the migratory capacity and dynamics of cells during inflammation. In summary, integrin affinity, clustering, trafficking and mechanosensing are regulated to give a specific response within a single cell (Figure 2).

### Classification of NK Cell Integrins by Beta Subunits

Traditionally, integrin β subunits have been used to generalize integrin functions; however, while such groupings have utility, they are frequently a convenience rather than a biological rule. Integrins can also be defined by other structural elements, such as the presence or absence of an αI domain, which is common to some β1 and β2 containing integrins and confers structural and functional similarities (31). Here we will examine the broad groupings prior to examining more closely the functions of the specific pairings related to cell behaviors ranging from migration to residency (Table 1).

**Table 1:** A list of natural killer cell integrins and their function in residency and/or cell migration.

**Alpha/Beta name**	**Alternate names**	**Binding partners**	**NK cells in tissue types**	**Migration types**	**Activity level**	**Residency associated signaling**
**β1; CD29**						
α1; CD49a	VLA-1	Collagen IV (high affinity), laminin (52–54)	Liver, lung, tonsil, uterus, skin, kidney, bone, spleen (55, 56)	ECM residency. Upregulated in cNKs that become trNKs (57, 58)	trNKs with high baseline activation. No CD62L (56)	CD69 expression—S1PR antagonism (59). CXCR3/6. Receptors for retention associated CXCL16 (60–62)
**β7**						
αE; CD103	–	E-cadherin (63–65)	Liver, lung, tonsil, gut, skin (mucosal propensity) (66)	Epithelial residency (67)	trNKs with less cytotoxicity than α1+ αE–. Can arise in α1+ cells in response to TGF-β (68, 69)	CD69 expression—S1PR antagonism TNF-α producing. CCL5, MIP-1β, and GM-CSF—recruitment and microenvironment remodeling (59)
α4	LPAM	MadCAM-1, VCAM-1, fibronectin (LDV) (70)	Liver, lung, tonsil, gut, skin (mucosal propensity) (66)	Epithelial residency (67)	trNK or cNK	CD69 expression—S1PR antagonism (59)
**β1; CD29**						
α2; CD49b	VLA-2	Collagen IV, III and I (low affinity), laminin, E-cadherin (1)	Absence coupled to α1 presence (55, 56). Blood and pan-tissue	Migration from circulation to tissue if α1-(55, 56)	cNK/trNK switchable (71)	Reduced TNFα production compared to α1+ α2– (59)
α3; CD49c	VLA-3	Collagen, laminin, fibronectin (1)	Blood and ECM	Adherence (24, 48)	cNK	–
α4; CD49d	VLA-4	Collagen, laminin, VCAM-1, MAdCAM-1, Fibronectin, ADAM (1)	Blood and ECM	Adherence (72–74)	cNK	–
α5; CD49e	VLA-5	Fibronectin (RGD), ADAM (1, 75)	Blood and ECM	Adherence (72–74)	cNK	–
α6; CD49f	VLA-6	Laminin, ADAM (1)	Blood and ECM	Adherence (72–74)	cNK	–
αV; CD51		Fibronectin (RGD), vitronectin (1)	Blood and ECM	Adherence (48)	cNK	–
α10;		Laminin, collagen (1)	Blood and ECM	Adherence (48)	cNK	–
α11		Laminin, collagen (1)	Blood and ECM	Adherence (48)	cNK	–
**β2; CD18**						
αL	LFA-1	ICAM-1,2,3, 4 (8)	Blood, lymph nodes, migration within diverse tissues (8)	Fast cell migration (76–78)	cNKs. Diapedesis, migration in lymph nodes, synapse formation (79)	Upregulated during inflammation to speed cell migration, scanning, and enhance diapedesis and synapse formation (79)
αM	Mac-1	ICAM-1, 4. Fibrinogen (8)	Blood, lymph nodes, migration within diverse tissues (8)	Cell migration	cNKs. Diapedesis, migration in lymph nodes, synapse formation (80–86)	αM/ αX low resident cells produce IFN-γ in the lung epithelium. Coupled to survival signals through CD27 (87)
αD	—	ICAM-3, fibrinogen, fibronectin, vitronectin, VCAM-1 (40)	Blood, lymph nodes, migration within diverse tissues (8)	Cell migration	cNKs. Diapedesis, migration in lymph nodes, synapse formation (6)	αM/ αX low cells produce IFN-γ and are coupled to high αE and α1 (88)
αX	CR4	ICAM-1, 4, fibrinogen, collagen (8)	Blood, lymph nodes, migration within diverse tissues (8)	Cell migration	cNKs. Diapedesis, migration in lymph nodes, synapse formation	During inflammation, αX β2 is reduced in resident bone marrow cells coupled with an increase in α1 (70, 89)

*Dark green, residency associated; Light green, non-residency*.

The β1 subunit can bind to 12 α subunits, and these heterodimers bind laminin, collagen, fibronectin, specifically LDV or RGD peptide domains, and VCAM1 (1, 90). Broadly, β1 heterodimers are often associated with tissue homing, providing a “bar code” for cells located in different kinds of tissue niches. Their functions contribute to cell adhesion, transmigration during entry or exit from tissues, and proliferation and survival (91). Specifically, α5β1 and α8β1 bind to RGD active sites in fibronectin, using a binding site at the interface between the α and β subunits. α4β1 and α9β1 bind the LDV peptide, which is structurally similar to RGD and contained within fibronectin, VCAM-1, and MAdCAM-1. Laminin and collagen binding integrins include α1β1, α2β1, α10β1, and α11β1, and their specificity is achieved through their use of the α A-domain. α3β1, α6β1, and α7β1 also bind laminin, but independently of the A-domain. Several non-β1 or β2 containing integrins also bind to RGD, LDV, laminin, and collagen (1). While β1 integrins are not leukocyte-specific, they are highly expressed on lymphocytes and play important roles in navigating tissue microenvironments. In particular, a1β1, a2β1, a4β1, and a5β1 are key components of tissue resident signatures that define residency, either by their expression or lack of expression, and are commonly found on NK cells. An overview of the main tissue resident integrins, comparing them to those found in circulatory cells or NK cells mediating cytotoxic killing, is found in Figure 1.

The β2 subunit forms heterodimers with 4 α subunits (αL, αM, αX, and αD), all of which bind ICAM-1 and fibrinogen and are leukocyte specific. β2 integrins have a conserved function in mediating leukocyte recruitment from circulation to the tissues [Figure 1; (8)]. They also help mediate target cell killing; in particular, αLβ2 initiates cell polarization through actin remodeling in the formation of an immunological synapse between NK and target cells, however αMβ2 is also found at the synapse (80–85). Nano-adhesions formed at the immune synapse (IS) in response to αLβ2 ligation are similar to those formed during NK and T cell adhesion to endothelial cells, diapedesis, or migration in tissues. The relatively small size of αLβ2 integrin signaling platforms speaks to their ability to remodel actin locally (2). Further, their catch bond function (41), in concert with other mechanosensitive molecules such as vinculin and talin (92), means that the movement of an individual cell can be fine-tuned by the spatiotemporal organization of these adhesion nodes. In the case of catch bonds, greater force applied to the cell results in a molecular response that increases the strength of binding. This is particularly important in the blood, where shear flow generated from blood pressure results in strong lymphocyte binding to endothelium, allowing the cells to subsequently crawl through to areas of infection in the tissues (93). As such, β2 integrins are important for the generalized ability of cells to migrate and communicate, and they are mostly associated with recruitment to tissues, rather than residency. While αLβ2 is most often used as a model for structural dynamics related to function (10, 12, 94–96), other integrins known to be important for NK cell locomotion and residency are also affinity regulated in this way. αDβ2 (CD11d/CD18) (6) and αMβ2 (CD11b/CD18, Mac-1) (86), both expressed by NK cells, adopt similar structural changes, which are closely regulated by the rearrangement of the MIDAS domain that is translated to I-domain movement (15, 97, 98). Integrin affinity is also regulated structurally in β1-containing heterodimers, which adopt bent-closed, extended-closed, and extended-open conformations which correspond to low, intermediate, and high binding affinity (75, 99). In some cases, the binding of integrin to ligand only occurs at a certain conformation corresponding to a given dissociation constant. α5β1 is an example of an integrin in which a single conformation can confer a low enough dissociation constant for binding, as only the extended open conformation binds to fibronectin (75).

Other β subunits further diversify the function of each heterodimer. In brief, the β4 subunit makes a heterodimer with α6, which binds laminin (1). The β5, β6, β8, and β3 subunits form heterodimers with αV, which bind the RGD motif in fibronectin (1). Finally, and of particular note in trNK cells, the β7 subunit makes a heterodimer with αE (CD103), which binds E-cadherin to form αEβ7, one of the main markers of tissue residency (100–104). β7 also makes a heterodimer with α4, which binds MAdCAM-1, VCAM1, and the fibronectin LDV peptide (1). In summary, integrins have different intracellular and extracellular ligands, and they are activated in response to a range of different signals. Diverse integrins must group together to enact whole cell change, or persistence in a given migratory state. Such specificities are intrinsically related to the tissue environment and the ligands found therein.

### Diversity of Integrin Ligands Within Tissue Occupied by trNKs

If the particular collection of integrins present on a cell membrane can be considered an “area code,” then it follows that the molecular composition of the environment defines the neighborhood. NK cells are located in non-lymphoid and lymphoid tissue environments, as well as in circulation in peripheral blood (105). In the blood, CAM family proteins, including VCAM, ICAM, and MadCAM, mediate leukocyte arrest and subsequent transendothelial migration between or through endothelial cells (106). During the process of extravasation, passage through the basal lamina, largely composed of collagens IV and VII and laminin, precedes entry to the underlying tissue. The non-lymphoid cells that comprise tissue also express CAMs, glycoproteins, and other integrin ligands, whereas the ECM is composed of a huge array of RGD- and LDV-domain containing fibers, for which different integrins have different affinities. Each tissue environment is unique in their composition of these binding sites for integrins on both cells and ECM.

ECM differs vastly between and within tissues and is composed of fibrous proteins including collagens and elastin, and glycoproteins such as fibronectin, proteoglycans, and laminin (107, 108). Collagens are the most abundant macromolecule in the ECM, and there are 28 different collagen subtypes which form fibers as tight homo- or heterotrimeric helices (108). Depending on the subtypes that make up the fibers, and the three-dimensional structures that they form, different binding sites become available for integrins. In addition, collagen fibers form supramolecular complexes with laminins, which act as bridging molecules and are composed of differing α, β, and γ chains that link collagen and integrins. In this way, 16 possible Y-shaped or rod-shaped laminin heterotrimers can be formed with different binding sites for integrins, and the differential combinations of these chains allows for great complexity between and within tissues (108). Fibronectin is another tensile molecule with many binding sites that facilitate linkage to collagen, integrins, and other components of the ECM, as well as to other fibronectin molecules. Fibronectin forms a homodimer that reveals cryptic binding sites when stretched, allowing dynamic control and two-way feedback between the ECM and lymphocytes (109–114). Integrin binding, particularly to RGD sites, promotes matrix generation, and adhesions formed within cells modify the structure of fibronectin fibrils in part by providing actomyosin-driven contractile force that is required for fibrillogenesis (112, 115–117). As fibronectin networks are built around cells *in situ*, it is important to try to hypothesize how these may affect trNKs entering an ECM-rich tissue then adapting to this environment.

CAM molecules are also present or absent depending on the tissue subtype and can be up- or down-regulated during inflammation and immunological memory responses. As primary ligands of β2 integrins, CAMs dictate functional responses depending on their density. Perhaps the best characterized of these responses is the arrest of rolling leukocytes followed by the control of tissue extravasation that occurs via ICAM-1-αLβ2 interactions. The local upregulation of ICAM-1 occurs in response to inflammation, enhancing the adhesion of leukocytes to blood vessel endothelium close to sites of infection (79). In NK cells, this interaction causes the polarization of lytic granules and actin rearrangement as a precursor to cell killing, while in cytotoxic T cells, ICAM-1/αLβ2 acts as a second signal for killing after TCR engagement with MHC in addition to functioning in adhesion (80–82, 84, 85, 118–124). Chemokines alter the nanoscale spatial arrangement of integrins, such that the presence of the CCR7 ligand CCL21 makes αLβ2 adhesion clusters condense, leading to faster T cell migration (76–78). As ICAM-1 and CCL21 are highly expressed in follicular DCs in secondary lymphoid tissue, their co-expression on T cells is linked with an increased efficiency of scanning DCs for MHC molecules and speaks to the multi-modal regulation of lymphocyte migration (31). As such, ICAM-1/αLβ2 is an excellent model to illustrate how interactions between the same CAM/integrin can lead to different outcomes dependent on the microenvironment. Other integrin-CAM combinations, such as αMβ2/ICAM-1, as well as those mediated by VCAMs, MAdCAM, and ALCAMs also have diverse roles dependent on the tissue microenvironment (125).

### Integrin Nanoclusters and Their Function in Lymphocyte Migration

Integrins in migrating fibroblasts group together in the lamellipodia at the leading edge, forming nascent adhesions of <100 nm in diameter (23, 126–129). Early adhesions, through the recruitment of cytoplasmic integrin adhesome proteins, coalesce into large clusters up to 1 μm in diameter at the basal membrane in the mid-body of the cell. These anchors allow cells to firmly adhere to their environment, forming large focal adhesions that are highly structured and turn over slowly (130). In contrast, lymphocytes operate a different integrin clustering program from fibroblasts owing to their need to move on a shorter time scale and quickly adapt to diverse environments. Most lymphocyte adhesion complexes are on the order of <100 nm in diameter and are only definable by super-resolution microscopy (2, 10, 17). While there are key differences between lymphocyte migration and the migration of larger, fibroblast-like cells, the model of tuning dynamic integrin-cytoskeletal linkages to promote adhesion and migration is conserved, and allows for different modes of lymphocyte migration or residency to occur in response to ligand specificity, intracellular signaling or tissue rigidity (131–134). In T cells, all αLβ2 clusters are <100 nm in diameter (2). These nanoclusters differ in their size and density depending on their location in a polarized cell membrane, namely the lamellipodia vs. the lamella, and have differential involvement of phosphorylated focal adhesion kinase (FAK) and Src family kinases. In 2D migration studies, the size of such nanoclusters, and the recruitment of active signaling intermediates, has been directly correlated with the velocity of migrating cells. Smaller, denser clusters with more phosphorylated kinases are associated with increased speed, indicating that integrins are regulated locally within signaling islands. Here, such active nano-adhesions were measured by tagging a single heterodimer in conjunction with signaling intermediates indicating active signaling (2). β1 integrins have been investigated in a similar way, albeit not in lymphocytes (135). The spatiotemporal regulation of αLβ2 integrin, and associated FAK and Src kinases works across length scales from single nanoclusters to groups of clusters that are translated into whole cell behavior providing dynamism to immune cells.

How diverse sets of integrins expressed on a single NK cell work together at this length scale is an open question. Mature NK cells co-express multiple integrins, including the β1 integrins VLA-4 and−5, and β2 integrin LFA-1 (72, 73, 136, 137). In migrating T cells, integrins are differentially localized along the front-rear axis based upon their conformation, with high affinity β1 integrins found primarily in the uropod, whereas LFA-1 is found in high-affinity conformation in the lamellipodia and mid-body focal zone, and low affinity conformation in the uropod (138, 139). T cell integrin spatial localization is linked to differential usage, which in the case of VLA-4 and LFA-1 includes mediating up- and down-stream mechanotaxis under shear flow, respectively (139, 140). Engagement of LFA-1 or VLA-4 to ICAM-1 or VCAM-1 leads to distinct signaling pathways that help shape these specified responses (140). Thus, at least in T cells migrating under flow, differential cellular localization of integrin species direct different functions in the same context, with the passive uropod acting as a windvane in LFA-1 mediated upstream migration, and the lamellipodia passively focusing downstream migration that is mediated by VLA-4 (139, 141). While not spatially defined on the nanoscale, there are also multiple mechanisms of integrin crosstalk, including both activating and inhibitory signals, that can be passed between integrin species (142–144). It should be noted that the example of VLA-4 and LFA-1 in this case is likely distinct from chemotaxis or 3D cell migration in a tissue microenvironment, in which integrins play different roles or may even be redundant (50, 134). Regardless, this example highlights the importance of using measurements such as integrin activation, signaling, and localization in addition to cell surface expression when defining the significance of integrin expression on cellular subsets.

While spatial patterns of single integrin localization have been described in some cases, species mixing to form complex, multi-integrin subtype clusters has not been investigated on the nanoscale. Both a4β1 and LFA-1 are recruited to lipid rafts upon T cell activation yet are found in distinct membrane patches in resting cells (145). While these studies were not performed with sufficient resolution to interrogate nanoclusters, it is conceivable that mixed clusters of integrins species are formed within lipid rafts. An alternative model is that individual integrins composed of only one heterodimer subtype form discrete nanoclusters, which has been shown for the T cell receptor and its adaptor LAT (146, 147). Segregation has also been found between active ligand-bound and inactive integrin β1 in conventional focal adhesions (148). Evidence that active and inactive integrins can co-cluster, but those that are ligand-bound or -unbound remain segregated, tells a more complex story (135). Communication between clusters in the membrane might be necessary to coordinate an appropriate migratory or resident phenotype and to employ dynamism between the two. Integrins move laterally in the membrane, or rather, the cell moves over anchored integrins, during mesenchymal or crawling-type migration and during reorganization for immune synapse formation (149). This cellular movement includes the centripetal flow of cortical F-actin and myosin-based contraction, which acts to strengthen high-affinity binding of LFA-1 to ICAM-1 (40) through mechanical forces.

While nanoscale information about the colocalization of multiple integrins has not been defined in NK cells, we do have insight into differential integrin expression associated with NK subsets. As we will see, α1 and αE integrin expressing NK cells represent a resident phenotype in multiple tissues. The absence or low expression of some integrins, including αM and α2, in such cells may be as relevant as the presence of others for cell and system behavior across length scales. High content and large field of view advanced super resolution microscopy and single particle tracking will be required to definitively dissect the relationship between integrin activation, localization, clustering and mixing on NK cells that are resident vs. migratory.

### Regulation of Integrins by Inside-Out Signaling From Chemokine Receptors

Chemokine receptors, the location of chemokine release or expression, and the corresponding cellular response are components of a cell's “area code” that are inseparable from integrin affinity, clustering and recycling. By having the capacity to rapidly induce integrin conformational changes and thus direct cell migration, chemokines are key regulators of the integrin-mediated response.

Chemokine receptors are grouped by their structure into four subtypes: CXCR, CCR, CX3CR, and XCR, with corresponding chemokine ligands for each (150). Functionally, chemokines can be grouped into “inflammatory/inducible,” such as CCL5 (RANTES), and “homeostatic/constitutive,” such as CCL19 and−21 (151). trNK cells, cNK cells, and developmental subsets of NK cells can be differentiated based on their chemokine receptor expression, which speaks to the interrelatedness of integrin and chemokine signaling in defining tissue residency and NK cell development. Key chemokines that dictate NK cell trafficking and function include CXCR1, CXCR3, CXCR4, CXCR6, CCR7, MIP-1α*/*β (macrophage inflammatory protein-1 alpha/beta, CCL3/4), RANTES (regulated activation, normal T cell expressed, and secreted, CCL5), and ATAC (activation-induced, T cell derived, and chemokine-related cytokine, CXCL1) (152).

Binding of chemokine to chemokine receptors results in the release of GPCR subunits into the cell that activate Rho, Rap, and Rac GTPases, which then modulate integrin affinity and clustering and actin remodeling (153). A generalizable model of the effect of how an arrest chemokine leads to rapid integrin activation includes the dissociation of Gβγ subunit, which diffuses into the cell to activate IP_3_-Ca^2+^ signaling and generate rapid intracellular calcium flux. Calcium and DAG in turn activate GTPases such as Rap1A which bind via adaptors to intracellular domains of integrins and generate intermediate conformation, thus priming them for ligand binding and subsequent strengthening of adhesion (154). In this way, chemokines work in concert with selectin-mediated tethering to rapidly activate integrins and switch modes of cell migration. It is important to note that while some chemokine signaling functions are conserved, such as the capability of CXCL12 to trigger an αLβ2 high affinity state in multiple lymphocyte subsets, downstream signaling pathways can be subject to variability and can be altered in the context of malignancy (155), speaking to the mutable nature of signaling islands.

### Inside-in Signaling—Integrin Trafficking and Recycling

Integrin outside-in and inside-out signaling describe a bidirectional interplay of mechanosensing, affinity and clustering by integrins and other receptors in conjunction with signaling intermediates. The process is also highly related to intracellular integrin trafficking. Far from being a simple process of recycling, integrin trafficking reveals that integrins also signal from intracellular vesicles (156). A pool of activated integrins in vesicles may be particularly important in cells that must remain dynamic after long periods of residency within tissues to respond to a new pathogen.

A universal measure of integrin activation is the local accumulation of active kinases such as phosphorylated tyrosine 397 (Y397) on FAK, and complexes of active FAK, integrins, and talin are present in endosomes (156). Without such endosomes integrin signaling cannot proceed, as they form part of the signaling axis emanating from the adhesome and resulting in Erk/AKT signaling. β1 integrins appear to be highly important in endosomal signaling and blocking Rab21-dependent endocytosis affects cell adhesion and migration. In particular, cells in which endocytosis of active integrin vesicles is impaired undergo anoikis (156). Further, integrins maintain their intermediate- or high-affinity conformation within vesicles through interactions with talin. Such active integrins in endosomes represent those that were previously engaged with ligands. Non-engaged integrins are constitutively endocytosed and recycled but lack the activation markers in vesicles described above. Clearly, such active integrin vesicles are important for recycling to the cell membrane to form new adhesions and must be especially important for fast-moving lymphocytes to generate new adhesions (157). Along with cell migration/adhesion receptors, endocytosed active integrins may cooperate with growth factor receptors, modulating their expression or trafficking to the membrane and ultimately the fate of the cell (158). While a role for endosomal integrin signaling has not been described in NK cells, defining how the activated cytoplasmic integrin pool changes in tissue resident cells could be a fruitful area for discovery.

## Integrins as Markers for Residency

Integrins and chemokine receptors are frequently used to describe differences between tissue resident subsets and circulating cells. As such, many of the key phenotypic markers that have been used with their CD nomenclature to describe unique tissue subsets are integrins. Below we will summarize these subsets with a particular focus on the integrins that define their residency and the implications of their expression.

### Integrins as trNK Markers in Mice

In mice, there are multiple populations of mature NK cells that are associated with unique tissue residency and are thought to undergo some, or all, of their maturation within these tissues. These subsets include conventional NK cells (cNK), which are found in spleen, blood, and bone marrow, thymic NK cells, trNK liver and skin cells, lung NK cells, and uterine NK cells (uNK) (159). By considering the expression and function of integrins on these specialized NK cell populations, we can speculate as to their relevance in establishing and maintaining NK cell residency and mediating function in different environments (Figure 1).

The full spectrum of NK cell developmental subsets is found in the bone marrow. Differentiation from common lymphoid precursors occurs there, and is followed by the exit of mature NK cells to the circulation and the seeding of perfused tissue such as the spleen (160, 161). However, the presence of highly specialized tissue NK cell subsets with unique transcriptional profiles suggests that earlier progenitors may also leave the bone marrow and settle in these tissues to undergo further differentiation (162–164). The presence of circulating common ILC and NK cell progenitors in peripheral blood also supports this model (165). Alternatively, the presence of innate-like B cells, macrophages, and mast cells in tissue that are derived from embryonic pre-hematopoietic precursors suggests that a similar pathway to the development of tissue resident innate lymphoid cells may also exist (166–168).

In the bone marrow, progressive maturation of NK cells is delineated in part by the upregulation of CD11b (Mac-1, αMβ2), and subsequent downregulation of CD27 (87). αMβ2 binds ICAM-1 to facilitate cell migration through the ECM and arrest in the blood prior to diapedesis, similarly to αLβ2, and strengthens binding to target cells during cytotoxic attack (83, 169). αMβ2 binds to 30 different protein and non-protein targets that are associated with migration and cytotoxic function, rather than residency (169). While the stages of human NK cell development are less frequently classified by CD27 and CD11b expression, upregulation of αMβ2 also marks mature circulating human NK cells (87, 170).

Notably, and irrespective of their origin, trNK cell subsets have differential expression of CD49a (VLA-1, α1β1) and CD49b (VLA-2, DX5, α2β1), which may reflect how integrins either direct precursors to, or retain mature cells in, these environments (55, 56). Further investigation into the molecular composition of ECM at different sites may also provide additional insight into why the expression of certain integrins is linked to distinct tissue resident phenotypes (107). For example, liver ECM is rich in type IV collagen, a high affinity binding partner for α1β1 (VLA-1) integrin (52–54). α2β1 binds less specifically to the GFOGER motif in collagens I and IV as well as GAOGER in collagen III (171, 172). Cell tracing experiments have shown that α1^−^α2^+^ positive (CD49b^+^) NK cells traffic to tissue sites via circulation, suggesting that their propensity for non-resident behavior may be mediated by the lower affinity of α2β1for collagen IV. The subset of liver NK cells that lacks α1 (CD49a) but expresses α2 (DX5^+^ or CD49b^+^) resembles splenic cNKs and are as such thought to be transient, whereas α1^+^α2^−^ NK cells are thought to be permanent residents of the liver (55, 56). These α1^+^α2^−^ liver trNK cells have low levels of αM but produce cytokines and are cytotoxic and are therefore not immature. They may represent a population that is derived after inflammation and have been linked to inflammation in the skin (55).

Liver trNK cells in mice are also functionally unique as they exhibit a higher level of baseline activation relative to splenic or liver cNK cells (56). They are larger and more granular, and express Ly49E, which mediates responses to liver-specific infections such as *T. cruzi* (173). Cytokine release is more efficient from these cells, TNFα, GM-CSF and IL-2 are all increased, and their high expression of TRAIL and FasL makes them better at inducing target cell apoptosis by alternative killing. Together, liver trNK cells may represent a resident subtype that acts both as a recruiter of other cells and an actively cytotoxic subtype. Of note, mouse liver NK cells represent a distinct lineage of trNK cells marked by a unique transcriptional profile and integrin repertoire when compared with cNK cells (174), adding another example to a body of literature that indicates that most trNKs develop *in situ*. In addition to their high expression of α1β1, which binds collagen IV strongly, CD69 is highly expressed in murine liver trNK cells and acts as an antagonist against the S1P receptor to prevent egress (175, 176). Together, this expression pattern works to maintain the tissue residency of mature NK cells (177).

In addition to liver trNK cells, the expression of α1 and the lack of α2 can also be used to define trNK cells in the uterus, skin, and kidney (56, 178), but not in other organs, such as bone marrow, lymph nodes, lung, spinal cord, pancreas, ementum, and peritoneal cavity. It is important to note that in the liver, uterus and skin, there are clearly defined populations of trNK cells and also cNK cells (55, 56, 179). In addition, the thymus and salivary glands contain NK cells, some of which have tissue resident properties. Thymic NK cells are cytotoxic, like cNK cells, but express the IL-7 receptor α chain (CD127) and arise from thymocyte precursors (56, 180–182). Uterine NK cells are also cytotoxic, like cNKs, but may arise from a separate developmental pathway, as they appear normal in T-bet-deficient mice (56). trNKs in the liver, uterus and salivary gland express both α1 (CD49a) and α2 (CD49b), whereas adipose, kidney and small intestine lamina propria ILC1 cells express only α1 and no α2 (183). Thymic NKs and circulating cNKs express only α2 (180). αE, which heterodimerizes with β7 to form αEβ7 (CD103), is expressed highly in salivary gland trNKs (184, 185) and is also present in other subsets such as lung and liver NK cells (186).

It remains unclear whether integrin expression patterns are pre-programmed, such that cells migrate to and remain in a given microenvironment, vs. being shaped in response to their local microenvironment. However, given that NK cells change their migration characteristics and integrin expression during development, during which they are exposed to diverse microenvironments, it is likely that the answer is a mix of both of these scenarios (187). Integrin β2 deficient mouse models show us not only that NK cells lacking β2 have impaired cytotoxic function, but also that they are developmentally impaired, failing to transition to maturity in all organs and demonstrating that β2 integrin expression is necessary for the homing of precursors to niches that can subsequently support their development (188). In contrast, β1 integrin deficient murine NK cells do not have impaired maturation or function, but have inhibited proliferation, a somewhat surprising but informative observation given the specific importance of β1 integrins in cell migration and association of their expression with tissue residency (189). Together these examples suggest that tissue seeding requires transcriptionally regulated integrin expression in mice. Conversely, differences in the strength of integrin-mediated adhesion to ECM between peripheral blood and lamina propria T cells can be recapitulated by short-term incubation of peripheral blood T cells with collagen or fibronectin (190). In addition, as discussed further below, there is evidence to suggest that tissue resident signatures can be generated or strengthened *in situ*. Human transplant studies have also demonstrated that markers of tissue residency, including αE (CD103) and α1 (CD49a), are acquired over time in a stepwise fashion in recipient T cells (65, 191). As the upregulation of their expression is linked to the acquisition of a transcriptional signature associated with tissue resident T cells, it is unclear what the local signals are that drive these changes, but they likely include chemokines, cell adhesion molecules, and ECM components.

### Chemokine Signaling to Integrins in Mice and Humans

Integrin expression and function are tightly linked to the expression and function of homing and chemokine receptors, which participate in inside-out signaling to regulate integrins and their downstream signaling networks. α1^+^α2^−^ liver trNK cells in mice highly express CXCR3 and CXCR6, both receptors for CXCL16, which is a chemokine expressed in liver sinusoids that mediates retention of NK and NKT cells in the liver (60–62). Liver trNKs also lack L-selectin (CD62L), which mediates rolling adhesion in capillaries and vessels (55). Together, these signals help generate a tissue resident phenotype by recruiting and maintaining NK cells in the liver. The specific up-regulation of molecules associated with homing and retention suggests that circulating mature NK cells can undergo phenotypic changes to become tissue resident. However, it is important to note that most literature indicates that trNKs develop through separate lineages from cNKs and establish residency phenotypes within tissues through development *in situ*.

trNK cells in the human lung are characterized by expression of CD69, a C-type lectin which antagonizes the receptor for S1P, which is a chemokine otherwise associated with egress from tissues. CD69 expression is a hallmark of tissue residency and has also been correlated with the co-expression of α1 and αE integrins, which contribute more to a distinct trNK phenotype compared to the presence of CD69 alone (59). As CD69 antagonism of S1PR prevents tissue egress, it is possible that CD69 upregulation precedes that of α1 and αE, such that circulating NK cells are trapped in the tissue and their residency is then strengthened by integrins. Further, collagen IV is rich in the basement membrane of the blood-gas barrier, so α1- expressing cells are more likely to localize here than the parenchyma. Their residency there may be reinforced by the inhibition of their egress from tissue following down-regulation of S1PR in response to CD69 ligation (59). While in many of these integrin-based mechanisms mouse and human trNKs share similarities, there are some key differences between species, and these often reside in the underlying transcriptional program. This is exemplified by Hobit, a transcriptional repressor that decreases expression of *S1PR1, KLF2* (which itself promotes CD69 expression), and *CCR7*, all of which promote tissue residency. In mice, Hobit directs a program of lymphocyte tissue residency that includes liver resident NK cells (192). In humans, Hobit is expressed more highly in circulating NK cells than in lymphoid tissue resident NKs (ltNKs) (70, 192, 193), but is also high in liver trNKs (194). The high expression of Hobit in circulating CD56^dim^ human NK cells, which correlates with Hobit-regulated genes, suggests that different pathways in humans and mice can dictate tissue residency (194).

In summary, and to continue the analogy, chemokine receptors constitute an area code for a GPS guidance system, whereas integrin expression may be more like a specialized tire set. Integrins allow cells to function in different kinds of environments; when they want to move around, they change their expression or alter the spatial positions of integrins in response to chemokines.

## trNK Integrin Expression in Humans

In humans, the enrichment of integrin ligands in given niches of organs often correlates with the expression of the integrins on immune cells, giving rise to diverse subsets of trNK cells found between organs as well as within them.

### Circulating NK Cells in Peripheral Blood

Human NK cells express αLβ2 (LFA-1) and αMβ2 (Mac-1) in circulation, in addition to α4β1 (VLA-4), α5β1 (VLA-5), and α6β1 (VLA-6) (72–74). For the most part, the expression of β2 integrins confers a specialized ability of circulating NK cells to adhere to endothelial cells in the capillaries, and mediate the formation of the immune synapse. β1 integrins are associated with mediating other functions, including tissue residency. The difference between the immune phenotype of leukocyte adhesion deficiency (LAD)-I and LAD-III in humans exemplifies the differential requirements for β1 and β2 integrins. LAD-I results from β2 integrin deficiency and affects exit of precursors and mature NK cells from the bloodstream as well as immune synapse formation (195, 196). LAD-III, on the other hand, is caused by mutations in kindlin-3 or the Rap1 GEF CalDEG-GEF1, which are conserved activators of β1 and β2 integrins, and thus leads to a broader clinical phenotype including bleeding disorders (197). Some integrin functions are conserved and/or compensated for in LAD-III patients, possibly by talin enabling α4β1 to maintain sufficient adhesiveness in the absence of kindlin-3 (198).

### Tissue Resident NK Cells in Humans

**Figure 3:**
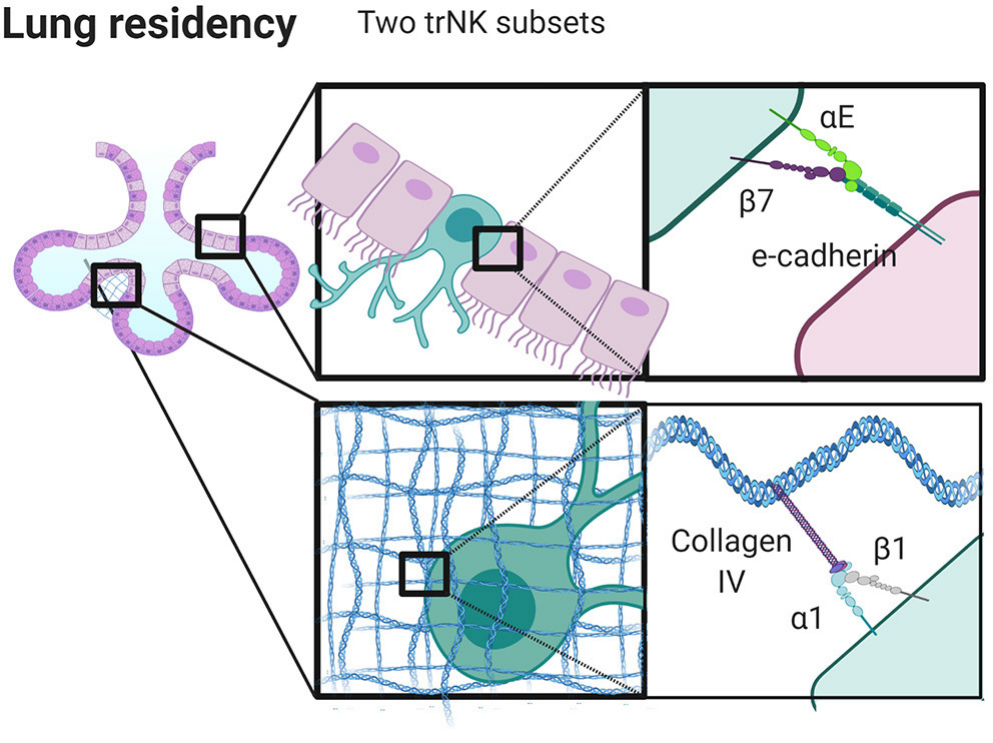
Integrins control niche specific trNK cell localization in the lung. trNK cells expressing αEβ7 localize to alveolar epithelium binding E-Cadherin, and α1β1^high^ trNK cells preferentially localize to the collagen IV rich basement membrane. Created with BioRender.com.

Tissue residency of both NK and T cells can be defined by a core transcriptional signature of genes aside from those that code for integrins. Decreased expression of *S1PR1* (S1P receptor 1) in mice (192, 199), and *SELL* (L-selectin), *RGS1* (regulator of g-protein signaling 1), and *KLF3* (Kruppel like factor 3) mark these populations in human NK and T cells (63, 200) in addition to an increased expression of CXCR6 on human NK cells (59). However, such non-integrin gene signatures also intersect with integrin profiles, as CD69^+^ α1 (CD49a)^+^ αE (CD103)^+^ trNKs in the human lung have non-integrin gene signatures that are distinct from those in the bone marrow and CD8 T_RM_ cells in the lung (59). α1 (CD49a), which binds to collagen IV, and αE (CD103) which binds to E-cadherin, are both markers of tissue retention in T cells (63–65). In addition, the “area code” that can direct cell trafficking through integrin ligand expression extends not only to organs, but to specific tissue niches within those organs including the human lung (Figure 3).

Taking the human lung as an example, the presence of αE likely not only signifies tissue specificity, but also spatial localization within tissue. There appear to be at least three distinct populations of tissue resident NK cells in the human lung, including CD69^+^α1^−^αE^−^, CD69^+^α1^+^αE^−^ and CD69^+^α1^+^αE^+^. These populations may reflect specific localization to specific regions in the lung. In human skin, α1 positive T cells are localized to the dermis, yet are seemingly unable to position within the epidermis. This finding has clinical implications as it affects the development of psoriasis, and it also demonstrates how sequestration of cells in tissue locales can occur based on integrin expression (67). Similarly, CD103^+^ (αE^+^) T cells are found within lung epithelium, suggesting that αE can be considered a marker of epithelial localization that correlates with expression of E-cadherin by epithelial cells lining the alveoli and basement membrane (201, 202). While the localization of trNK cells in the lung has not been well-defined, it is likely that the distinct populations found here are also spatially distinct, with CD103 marking a population found in the epithelium (discussed further below and summarized in Figure 3). These cells may undergo pre-programmed fate decisions, anchoring themselves into long term tissue residency as they undergo maturation. Alternatively, a plethora of immunological stimuli combined with the composition of the microenvironment could produce a heterogeneous population of cells which can then adapt to become resident in different locales in part through modifying integrin expression (59).

### Relating Phenotypic to Functional Differences in the Lung

When we consider the differential expression of α1 and αE in lung, we can speculate as to the relative roles of these integrins on NK cells (Figure 3). The human lung is a mucosal environment that is frequently exposed to pathogens and where tissue resident lymphocytes are crucial for managing acute and chronic threats. Tissue resident CD8^+^ T cells have a unique transcriptome and their frequency is correlated with lung cancer clinical outcomes (203). Unlike T cells in the lung, trNK cells have poor lytic function when challenged with tumor target cells *ex vivo*, but maintain their ability to kill virally infected cells (59). Such seemingly hyporesponsive cells also have the ability to produce inflammatory cytokines and their lytic function can be restored by stimulation with IL-15 (59). α1^+^αE^+^ positive cells are less cytotoxic than α1^+^αE^−^ cells, but both subsets produce comparable quantities of TNF (59). This suggests that αE may play a functional role in pushing cells away from a cytotoxic phenotype, potentially through its interaction with E-cadherin, although the functional role of CD103 is less well defined than that of CD49a (202, 204). The expression of α1 integrin suggests plasticity, as α1 expression can be induced by the presence of IL-15 (59, 205). Further, α1^+^ lung trNK cells upregulate perforin, granzyme B, Ki67, and CCL5, markers of greater functional capacity, to a greater extent than cells that are α1 integrin negative in the lung (59). While blocking CD49a abrogates cell adhesion and migration on collagen IV *in vitro*, cells fail to migrate on E-cadherin, and CD103 does not participate in collagen binding or cell migration (202). Together, this raises questions as to the relative contributions of α1 and αE and how they are regulated. Is α1 integrin internalized, but remaining in pools ready for quick use? Does it remain on the surface, and change its role in the presence of αE integrin as a part of inter-integrin inside-out signaling? It is important to note that trNK cells in the lung express significant amounts of CCL5, MIP-1β, and GM-CSF, which are all chemokines involved with modifying the microenvironment, shaping cell migration and recruiting other immune cells (59).

Finally, α1^+^αE^+^ trNK cells in the lung also express less L-selectin than α1 or αE negative cells. Expression of S1PR5, which is a receptor for S1P, a chemokine known for the induction of egress from lymphoid tissues, is also low in these cells. CD69 antagonizes S1PR1 by causing its removal from the cell membrane and its internalization, suggesting that the expression of CD69 and lack of expression of S1PR5 helps mediate retention of these cells in tissue (206). In addition, α1^+^αE^+^ trNK cells in the lung express more β1 integrin, which is associated with adhesion rather than migration, likely further contributing to the tissue resident phenotype (59).

### Liver α1^+^ Cells Represent a Distinct Lineage of trNKs

**Figure 4:**
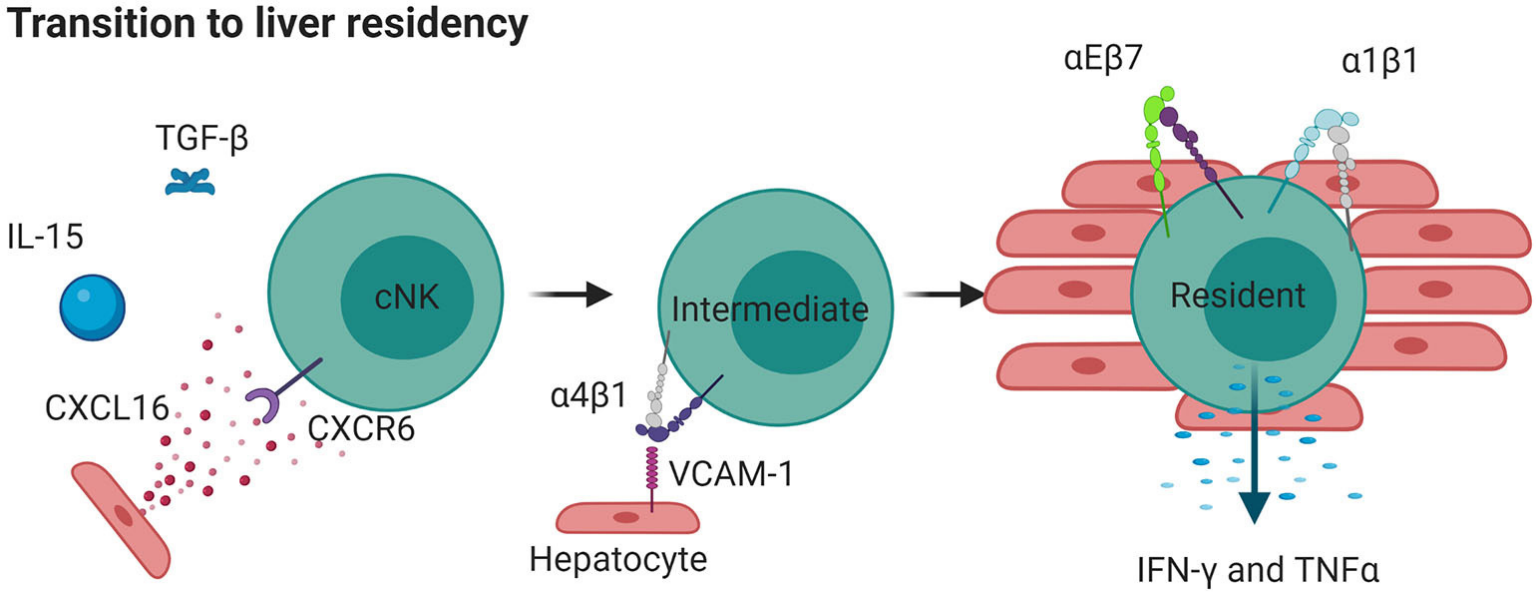
Liver resident NK cells can originate from cNKs. IL-15 and TGFβ released during inflammation cause cNKs to upregulate CXCR6 which detects CXCL16 in the liver. This results in more α4β1, moving cNKs from blood to liver, where they upregulate αEβ7 and α1β1 and become tissue resident. Such cells are less directly cytotoxic but produce high levels of cytokines IFNγ and TNF. Created with BioRender.com.

A population of trNK cells that is partially defined by expression of the integrin CD49e (CXCR6^+^ or CD49e^−^CD56^bright^) has been clearly identified in the human liver (207, 208). Unlike conventional NK cells, liver α1^+^ NK cell differentiation requires T-bet, but not EOMES (186). EOMES and T-bet, both transcription factors that can define the maturation of NK cells, are present in mature NK cells of the periphery and uterus, whereas the EOMES^−^ population in liver appears to be rare in humans, although a minor EOMES^−^ population expressing α1 has also been found to produce TNF during pregnancy (209, 210). This liver resident NK cell population is found in the liver tissue itself, but not in afferent or efferent hepatic blood or peripheral blood and represents an equivalent α1^+^α2^−^ population to that found in mice (56, 174). The presence of liver trNK cells in adult but not fetal liver suggests that these are a subset of memory NK cells that arise due to environmental stimulus, again emphasizing the potential for phenotypic switching suggested in the lung. This adds to a large body of evidence describing the development of trNK cells from precursors within tissue instead of from mature NK cells that seed tissue from the blood. Complexifying the situation, there also exists an EOMES^high^ population of trNK cells detected in the liver over timepoints spanning 13 years (68). However, unlike the population described above, which seems to be a separate lineage that remains resident, the EOMES^high^ population seems to be derived from cNK cells in the blood (68). It is thought that EOMES^low^ cNK upregulate EOMES in response to cytokines that likely include IL-15 and TGF-β, resulting in reprogramming and the detection of many liver tissue residence molecules such as integrins α1 and αE (68, 69). These cells likely first upregulate CXCR6, which senses CXCL16, which is highly expressed in the liver especially during an inflammatory event. CXCR6 activates integrin α4β1 (VLA-4), which can enhance the recruitment of cells to the liver through binding to VCAM-1 in mice [Figure 4; (211)]. Upon seeding of tissue, trNK cells may upregulate EOMES and other related retention signals, including α1 and αE, to generate a tissue resident phenotype [Figure 4; (71)]. Therefore, the function of trNK cells, whether derived from blood or *in situ* maturation, may be to provide a version of adaptive immunity that can be shaped by integrin function in response to inflammation or infection. As with the equivalent α1^+^α2^−^ mouse subset, the human liver trNK subset produces IFNγ and TNF to a higher degree than conventional NK cells, and are less cytotoxic based on their inability to degranulate efficiently and their low levels of perforin (186). They are highly proliferative in *ex vivo* culture and have KIR expression that suggests clonal origins, suggesting that these are memory NK cells (186). Regardless of their origin, due to their distinct transcriptomic profiles from conventional NK cells it is likely that liver trNK cells develop or mature in the liver at least partly in response to environmental cues.

Comparing the localization of trNK cells in the liver with those in the lung, the expression of α1 means that these cells also express β1, implying that they use this receptor functionally to bind to collagen that is present at high levels in the liver parenchyma (186). In the lung, the basement membrane of the blood-gas-barrier is rich in collagen IV, which is not present in parenchyma (59). αE uses binding to E-cadherin to localize T cells to epithelial tissues in the lung (212). As such, this is another example of how the expression of integrins can result in different localization the same tissue, dependent on the binding partners that are available. This behavior is replicated in double positive α1 and αE trNK cells in the lung (59). The localization of liver α1^+^αE^+^ trNK cells is likely to also be biased toward E-cadherin and collagen IV rich regions, and together this highlights the specificity and degree of residency that is achieved by the plastic expression of integrins together with differential expression of their binding partners in the tissue microenvironment. By tuning their killing and cytokine release abilities, which can also be correlated to their integrin expression, such cells are likely able to respond to infections common to these tissue regions.

Both α1 and αE, therefore, are associated with cell stopping and retention, rather than migration, and as such enable tissue residency. As described previously, expression of other integrins, such as αLβ2 and αMβ2, generate a more migratory phenotype within lymph nodes for scanning, or to aid in diapedesis into tissues from HEVs. Together, this points toward a shared transcriptional code for trNK cells, which can be further diversified depending on the organ and the precise tissue locale within the organ and is distinct from that of CD8^+^ T_RM_ cells. This speaks to the combination of predetermined function and plasticity shown by NK cells that may either be programmed to become tissue resident or may respond directly to the combination of signaling molecules and other cues, such as mechanotransduction, from their environment.

### Integrins and Tonsil Tissue Residency

The tonsil has been well-studied as a site of NK cell development, and in addition to other secondary lymphoid tissue represents an important site of human NK cell development and residency (162, 163, 187, 213). This is in part due to the presence of an early NK cell precursor defined by the expression of integrin β7, which is thought to seed tissue after exiting the bone marrow. This precursor can also be found in peripheral blood expressing L-selectin, suggesting that expression of L-selectin helps recruit these precursors to tissue before being subsequently down-regulated (162, 163).

Tonsil resident cells are thought to include intraepithelial ILC1 helper cells that have an integrin expression profile similar to CD8^+^ T cells and are characterized by their expression of NKp44, αE integrin, and CXCR6 (88). While the etiology of ILCs in tonsil has since been debated (214), the tissue resident aspect of the phenotype of ieILC1s is conserved between multiple tissues. While similar in many respects, and likely belonging to the same lineage, ieILC1 cells are slightly different from trNK cells, having similar abilities to produce TNFα and IFN-γ but less cytotoxic capacity (88). Generally, cNKs do not highly express IL7Rα, and can be differentiated from ILC1s on this basis. However, some trNK cells, including those in the siLP, thymus and elsewhere, actually do express the IL7R, further complexifying their discrimination from ILCs (180, 215).

Unsurprisingly, cNK cells in the tonsil that express αE also express high levels of β7, the heterodimeric partner of αE, yet they additionally express high levels of α1 and lower levels of αM and αX (88). Like αM, αX forms a heterodimer with β2, and overlaps in many of its functions of adhesion, migration, and diapedesis in activated cells. As in the liver (216) expression of CXCR6 is high, and expression of CCR9 is low on α1^hi^, αE^hi^, αM^lo^, αX^lo^ NK cells from the tonsil (88, 216). Interestingly, NEDD9 (CasL), a protein associated with inside-out integrin signaling via its interaction with FAK, is also very highly expressed in these cells, suggesting that adhesion regulators may also mediate integrin functions associated with residency (88, 140, 217). In addition to its well-described role in liver residency, CXCR6 is associated with specificity for epithelial tissues, including the airways of the lung (61, 218, 219). Similarly, the CCR9 ligand CCL25 induces lymphocyte entry to the small intestine (220–223). α1, as previously discussed, has high affinity for collagen. Together, α1^hi^ αE^hi^ αM^lo^ αX^lo^ NK cells reside close to the epithelium in the tonsil and seem specialized to produce IFN-γ on demand. The specific integrin profile of tonsil resident NK cells is therefore linked both to the specific expression of chemokine receptors and adaptors.

In other secondary lymphoid tissue, CD69 and CXCR6 expression define a lymphoid tissue trNK subtype (ltNKs) that is found in isolated human secondary lymph nodes (70). In common with liver trNK cells, ltNKs downregulate S1PR1. As CD69 antagonizes S1PR1 signaling, this is a strong signal for remaining in the same locale, as S1P is high in the blood and S1P gradient disruption directs trafficking decisions (175, 176). CD62L (L-selectin) is also downregulated in secondary lymphoid tissue, leading to increased tissue retention. CCR7, again, is strongly associated with sensing CCL21 and CCL19 in the blood and is downregulated in ltNKs (66, 70). Together, these signals act to retain NK cells in lymph node and can be tuned in response to infection. In addition, in infected lymph nodes, increased CD49b-mediated interactions with collagen confine NK cell migration to restrain them near sites of infection, and CD49b cross-linking enhances NK cell cytokine production (224, 225). CD49b (DX5) positive NK cells also home to lymph node under homeostatic conditions and migrate on collagen (224, 226). While there is debate about the relevance of integrin-mediated migration in lymph nodes (50, 134), intravital imaging clearly reveals roles for both β1 and β2 integrins in certain settings in mouse lymph node (224, 225, 227).

### Endometrial trNKs Are Defined by Their Expression of α2 Integrin

**Figure 5:**
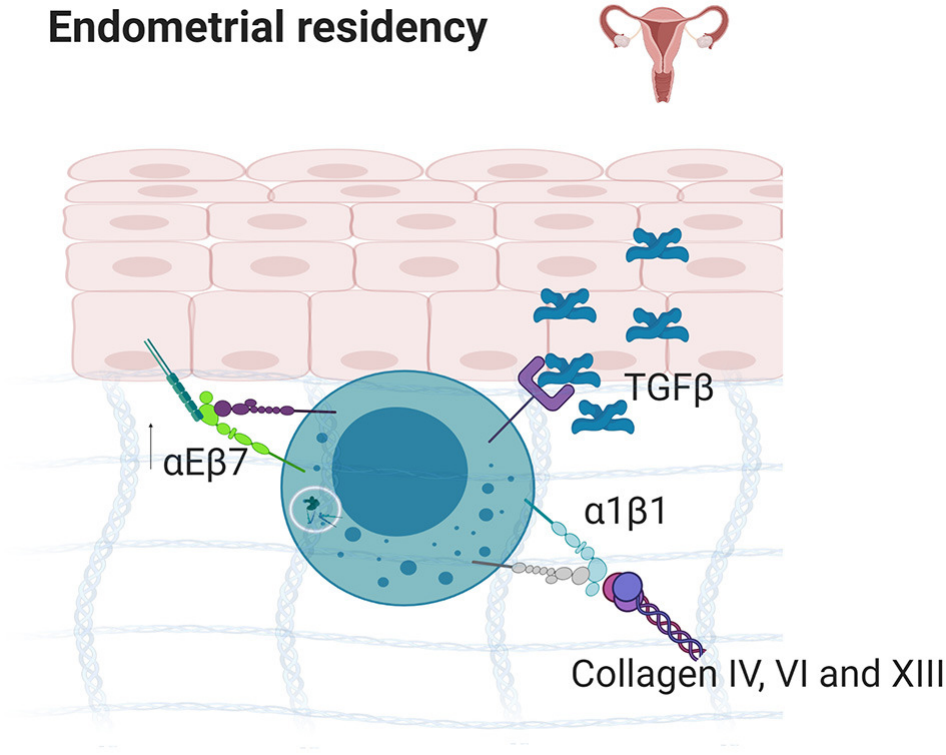
Endometrial residency. TGFβ secretion in the endometrium results in the upregulation of α1β1 and αEβ7 which bind to E-Cadherin and collagens IV, VI, and XIII, resulting in strong tissue residency. Created with BioRender.com.

Decidual and uterine NKs are important for successful gestation and are the predominant lymphoid cell type during the first trimester of pregnancy (228). In particular, dNKs are CD56^bright^ and α1 integrin positive, are programmed to promote neoangiogenesis, tissue remodeling and placenta development, and may originate from cells that develop *in situ* in the uterus or traffic there from lymph node or bone marrow (229). dNK cells share the ability to secrete TNF and IFNγ with ILC1s, while also having cytolytic capabilities. In this environment, stromal release of TGFβ is a driving signal that induces αE expression, suggesting that α1 positive cells arise through development and that the expression of αE arises in response to local cytokine release to help promote residency [Figure 5; (209, 230)]. In mice, two subsets of dNK cells are present, both expressing EOMES and α1, but one is α2 negative and the other is α2 positive (209). A third population, negative for α1 but positive for α2 and EOMES, is also present in the uterine tissue, and represents conventional NK cells (209). As is the case in the intestine, αE expressing trNKs in human endometrium can be further dissected into two populations based on their expression of NKp44, both of which are α1^+^α2^+/−^ (209). Interestingly, the expression of α1 is correlated with distinct function, as a1^+^ dNK have increased TNFα production relative to a2^+^ cells. These phenotypes suggest that the *in situ* development of these types of cells from precursors of both peripheral and medullary origin, highlighting the plasticity of NK cells to transition into trNK cells.

### Bone Marrow NK Cells and Their Tissue Resident Integrin Signature

In the adult bone marrow, HSCs maintain residency for life, but it is unknown as to whether mature NK cells found there develop *in situ* or come back and form a resident phenotype after being in circulation or developing elsewhere (164). Recently, a subset of mature trNK cells with a unique transcriptional program including integrin and chemokine receptors was defined in the human bone marrow (70). These bone marrow trNK cells have a common tissue resident phenotype marked by expression of high levels of CD69 and CXCR6 (70). However, they express more S1PR1 than lung trNKs, possibly speaking to greater plasticity of trNKs in the bone marrow. Finally, bone marrow trNK are also EOMES^high^Tbet^low^, similar to trNK cells in the liver, suggesting that these are true NK cells that are developmentally separate from ILC1s.

**Figure 6:**
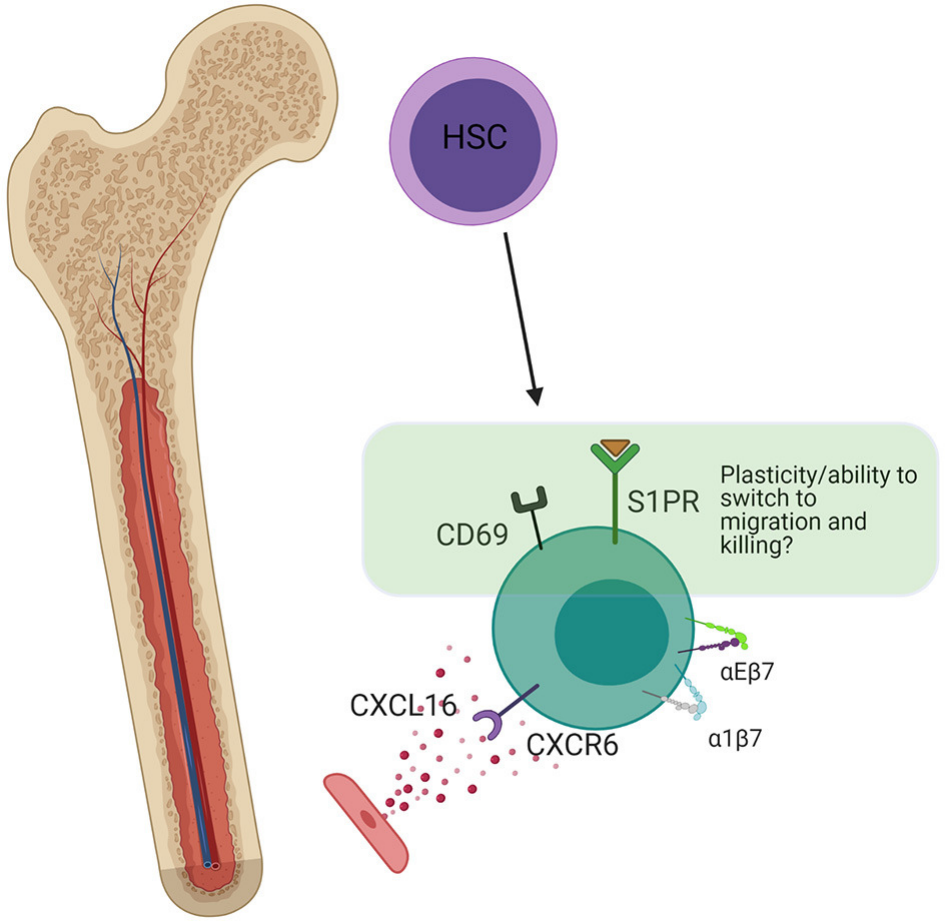
Bone marrow residency. Bone marrow resident NK cells are thought to develop *in situ* from HSCs. The presence of a small amount of S1PR (not present in trNKs in mucosal tissues), indicates the potential for plasticity and switching into a non-resident phenotype. CD69 is still present, which antagonizes S1P signaling, but at lower levels than in the lung. CXCR6 strengthens long-term residency through upregulation of α4β7 and αEβ7. Created with BioRender.com.

In addition to CXCR6, CD69 and aE, a generalizable tissue resident phenotype can be defined by the reduced expression of integrins associated with migration, diapedesis and/or scanning in lymph nodes, and synapse formation. The reduced expression of αX, α5, and β1 on human bone marrow NK cells is such an example, as αXβ2 is associated with cell migration and, in monocytes, is responsive to ligands that are upregulated during inflammation to enhance scanning, migration, and activation (70, 89). α5 couples with β1 and is also absent on human liver trNK cells (207). Distinct from the salivary glands, lung, and skin epidermis, human bone marrow trNK cells have reduced expression levels of β7, which can couple with either αE or α4 (70). The former binds cadherins, while the latter binds fibronectin or VCAM-1. In other organs, αEβ7 and α4β7 mediate cell localization close to the epithelium, the entry point for many pathogens. This difference is an example of one of the ways that trNKs in mucosal epithelial tissues such as tonsil, gut, skin, and lung, are phenotypically different from those in non-mucosal tissues including spleen, liver, and bone marrow. In terms of integrins, the main difference is that trNK cells rely on high α1 and αE expression in mucosal tissues, which appear to be reduced in non-mucosal trNK cells (66). As high α1 and αE are also accompanied by TGF-β imprinting, this points toward a propensity for some NK cells to switch to a resident phenotype upon entry into the mucosal tissue (57, 58). In the BM, it is less clear that NK cells do this, and it is possible that trNK cells here preferentially develop *in situ* through distinct developmental lineages (Figure 6).

### αE and α1 Positive Cells Operate a First Line of Defense in the Intestine

In the fetal intestine, a population of EOMES positive, αE positive, and IL7Rα (CD127) negative trNK cells dominates and is present at a stage of development that allows them to directly and indirectly respond to viruses during a time when a newborn is vulnerable due to a developing immune system (183, 231). These innate cells provide a first line of defense while canonical adaptive immune cells are still developing. Given foreign challenges, the tissue residency of NK cells in the fetal intestine is very important and is based on their expression of integrins. It is thought that this population is replaced by an EOMES^+^ T cell population later in life that similarly functions to protect this unique site (183).

**Figure 7:**
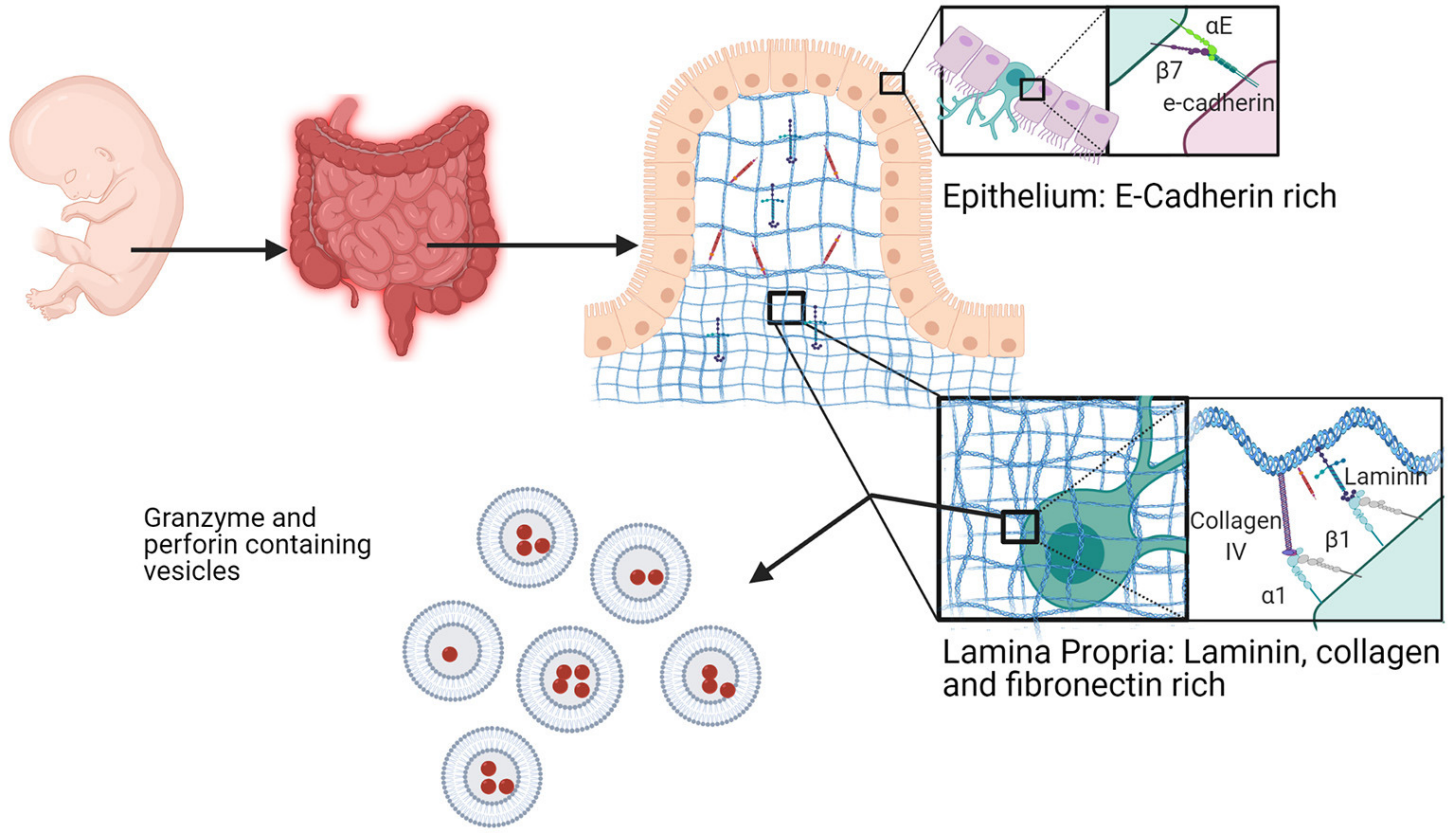
Fetal intestine resident NK cells. NK cells in the fetal intestine exist in the epithelium and the lamina propria. In the epithelium, trNKs express high αEβ7 to bind to E-cadherin at cell junctions. trNKs upregulate α1β1 in the lamina propria, where there is high expression of fibronectin, collagen, and laminin. Fetal intestine trNK cells (of either subtype) have high levels of granzyme- and perforin-containing vesicles. Created with BioRender.com.

In terms of integrins, trNKs in the lamina propria, the connective tissues at the base of the epithelium, are distinct from those in the epithelium. The lamina propria is an elastic, collagen III rich environment, which gets progressively denser in collagen moving away from the epithelium toward the muscle. It is an area rich in fibroblasts and adipocytes, which produce fibronectin, laminin, and collagen III (232). Within the epithelium, there are both CD45^+^ IL7R^+^ (CD127^+^) ILCs and CD45^+^ IL7R^−^ NK cells. Again, trNK cells in the fetal intestine are much better at producing granzyme B and perforin than cNK cells and CD69 positive cells (183), in the case of both epithelial and ECM resident subtypes (Figure 7). Neonatal trNK cells are much more potent, in these regards, relative to their adult counterparts, most of which are αE^+^ and/or α1^+^ and CD69^+^. Notably, the cells that persist into adulthood are NKp44^+^αE^+^CD69^+^, whereas the NKp44^−^αE^+^CD69^+^ populations present in infancy die off (183). A failure of this reprogramming may relate to the development of autoimmune disorders (233–235).

## The Future of trNK Study: Linking Single Molecule and Clustering Behaviors With Single Cell and Population Level Behaviors

Integrins are important for tissue residency between organs and within them, and most of what we know is based on their upregulation or downregulation in terms of expression. By thinking about the upregulation of αE in mucosal trNKs, shown in T cells to be a result of TGF-β secretion, we can turn our attention to plasticity, tracking cells as they develop and move. Human organoid systems combined with DNA barcoding and/or long term microscopy imaging and quantification may now help us to understand which cNKs then go on become trNK cells, compared to which trNK cells arise directly from developmental precursors or immature NK cells. We know that the microenvironment of a given sub-tissue level environment, for example the epithelium in the alveoli at the blood-gas barrier, is distinct, and that this results in a program of distinct integrin expression. Whether trNK cells are hardwired to express this integrin program before they localize and survive in these tissues, or whether cNKs switch to this program upon entry, is an area for further study. Further, while most work so far has been focused on expression levels of integrins, their spatial localization as well as their affinity state dictates the downstream effects of their ligation. To truly understand the complex interplay of multiple integrins functioning in concert, it will be of value to carefully dissect the expression, localization and function of multiple integrins on a single-cell basis in a heterogeneous population within the microenvironment.

### Cross Length-Scale Integrin Biology: Linking Nanospatial Phenomena to Heterogenous Cell and Population Behavior

The behavior of nanoscale integrin interactions is as important as their expression. Clustering is a phenomenon that has been shown conclusively for αLβ2 integrin in T cells which form very small integrin-mediated adhesions in the membrane (2, 131). The size, density, and number of molecules per nanoscale cluster is correlated with the number of phosphorylated FAK and Src family kinase molecules in individual adhesions, and the speed of the cell and its actin flow rate, despite different cells having roughly the same amount of total integrin (2). While informative, most work on the nanoscale composition of integrin adhesions has been focused on a single integrin subtype, whereas the co-clustering of multiple integrins remains relatively uninvestigated. α1 and αE are often upregulated together in trNK cells, and an open question is whether such integrins form nanoclusters in the same way as αLβ2 in T cells and whether both integrin species are found in the same cluster. If this was the case, their downstream signaling partners might work together to produce specific cell behaviors during the switch from tissue resident to cytotoxic phenotype upon recognition of a pathogen. If α1 and αE form separate clustered islands, as seen for other molecules (236), this could indicate competition for ligands that could affect cellular responses. In the case of αEβ7, β7 also binds α4, and both are present in NK cells. Whether this results in αE outcompeting α4 for β7 binding or construction in the ER, or whether both are produced at similar amounts and are regulated spatiotemporally in the membrane is unknown. Such a phenomenon would represent an example of spatiotemporal integrin competition in response to changing conditions of the microenvironment.

trNKs exist in a primed state, where they are simultaneously long-lived and stable, but are ready to switch at a moment's notice to migratory, cytotoxic or recruitment-type behaviors. Whether the integrins that are expressed by these cells mirror this stable but also dynamic behavior is an open question, directly related to cross-length scale biology. Some integrins may act as seeds for signaling, establishing themselves in the membrane prior to recruiting other integrins. Secondary, recruited integrins might add dynamism and competition within integrin clusters that allow cells to switch their adhesive behavior, and could be addressed by using single particle tracking to watch the interaction of two integrins within the same cells. The use of a growing range of small molecule tags (237), namely frankenbodies (238), SunTag (239), and MoonTag (240), and cell permeable bright stable organic dyes (241) may allow this type of nanoscale investigation to be undertaken.

Previously, we have discussed how β1 integrin heterodimers are generally more useful for invoking residency when compared to β2 integrins which induce migratory and/or active cytotoxic phenotypes. On a single molecule level, this could be due to their reduced propensity to form catch bonds in response to mechanical stress across the molecule. This may speak to β1 integrins having different roles as single receivers, rather than as migration-inducing mechanotransducing molecules. One experimental way to address this would be to measure the tension across the integrin using new molecular tools (242) to determine how differential force affects the localization of downstream activated intermediates. Such intermediates might include phosphorylated FAK, Src kinases and phosphatases, which could be imaged by live cell microscopy in NK cells operating in different microenvironments. Such microenvironments are intricate, and new data reveals different integrin adhesome programs that arise in communication with these heterogeneous locales (243). Investigating the tension across molecules, while also measuring the behavior of cells in populations, is another way to link function to behavior using new technologies.

In migratory NK cells, as in migratory T cells, β2 integrins form adhesions which must be turned over, either removed from the membrane and degraded or recycled, very quickly due to the speed of migration of the cell over these anchor points (2). Adaptor proteins and intermediates are therefore likely to be tailored to different rates of transience in the membrane, where high transience is associated with the recruitment of Src family kinases, FAK (2) and Crk (244) in diapedesis and in response to infection. Resident phenotypes associated with high β1 incidence in the membrane may be associated with affecting the production of inhibitory molecules that further promote residence, such as CD69 that inhibits S1PR receptors to prevent egress from tissue (245). This highlights the ability of longer-lived β1 adhesions to induce different behaviors to transient β2 adhesions based on their recruited signaling intermediates. Together, linking nanoscale/single molecule behavior of integrin αβ heterodimers in nanoclusters at the membrane and inside cells, with individual cell behavior and collective cell behavior in a heterogeneous physiological system is a wider goal of the field. New technologies such as automated super resolution microscopes that scan the plate and image cells based on their shape enable matching of nanoscale molecular organizations with cell movement and behavior (246). Machine learning increases the number of parameters for automated cell selection prior to imaging and enables the ability to segment thousands of cell behaviors post-acquisition and match them to nanoscale phenomena. These technological advances will enable us to link integrin adhesion behaviors on a molecular level to single cell behaviors and population behaviors and are primed to give us access to the molecular basis of NK cell tissue residency, migration and development.

## Conclusion

With a greater understanding of the importance of trNKs within tissues comes a better appreciation of the cross length-scale role of integrins in their regulation. In multiple tissues, and in most cases, trNK cells arise from developmentally distinct lineages but can also derive from cNK cells that transit from the blood after inflammation in response to integrin and chemokine receptor signaling. Further, we highlight their plasticity, as trNK cells may change their integrin expression to become different upon viral infection and/or long-term residency. Integrins have multiple roles, therefore, in residency and for tuning a functional response for subsequent challenge. This makes sense as integrins are involved in adhesion, migration, and communication with both cells and ECM. Embracing heterogeneity by correlatively imaging diverse populations of NK cells in stromal or organoid based systems will help to further link the functions of nanoclusters of specific integrins that operate a tailored program within single cells to their population-level functions.

## Author Contributions

MS and EM contributed equally to writing and editing. All authors contributed to the article and approved the submitted version.

## Conflict of Interest

The authors declare that the research was conducted in the absence of any commercial or financial relationships that could be construed as a potential conflict of interest.
